# Depression and food insecurity among patients with rheumatoid arthritis in NHANES

**DOI:** 10.1186/s41927-021-00236-w

**Published:** 2022-02-02

**Authors:** Qian Cai, Jacqueline Pesa, Ruibin Wang, Alex Z. Fu

**Affiliations:** 1 Janssen Scientific Affairs, LLC, 1125 Trenton Harbourton Road, Titusville, NJ 08560 USA; 2 Harvard TH Chan School of Public Health, Cambridge, MA USA; 3 Georgetown University Medical Center, Washington, DC USA

**Keywords:** Rheumatoid arthritis, Depression, Food insecurity, Social determinants of health, NHANES

## Abstract

**Background:**

Social determinants of health (SDH), including food insecurity, are associated with depression in the general population. This study estimated the prevalence of depression and food insecurity and evaluated the impact of food insecurity and other SDH on depression in adults with rheumatoid arthritis (RA).

**Methods:**

Adults (≥ 18 years) with RA were identified from the 2013–2014 and 2015–2016 National Health and Nutrition Examination Survey (NHANES). Depression was defined as a score of ≥ 5 (mild depression: 5–9; moderate-to-severe depression: 10–27) using the Patient Health Questionnaire-9 (PHQ-9). Food insecurity was assessed with the 18-item US Household Food Security Survey Module. Adults with household-level marginal-to-very-low food security were classified as experiencing food insecurity. The prevalence of depression and food insecurity among participants with RA were estimated. Weighted logistic regression was used to evaluate the association between depression and participants’ characteristics including SDH. Penalized regression was performed to select variables included in the final multivariable logistic regression.

**Results:**

A total of 251 and 276 participants from the 2013–2014 and the 2015–2016 NHANES, respectively, had self-reported RA. The prevalence of depression among these participants was 37.1% in 2013–2014 and 44.1% in 2015–2016. The prevalence of food insecurity was 33.1% in 2013–2014 and 43.0% in 2015–2016. Food insecurity was associated with higher odds of having depression (OR 2.17, 95% CI 1.27, 3.72), and the association varied by depression severity. Compared with participants with full food security, the odds of having depression was particularly pronounced for those with very low food security (OR 2.96, 95% CI 1.48, 5.90) but was not significantly different for those with marginal or low food security. In the multivariable regression, being female, having fair/poor health condition, any physical disability, and ≥ 4 physical limitations were significantly associated with depression.

**Conclusions:**

In adults with self-reported RA, the prevalence of depression and food insecurity remained high from 2013 to 2016. We found that depression was associated with SDH such as food insecurity, although the association was not statistically significant once adjusted for behavioral/lifestyle characteristics. These results warrant further investigation into the relationship between depression and SDH among patients with RA.

**Supplementary Information:**

The online version contains supplementary material available at 10.1186/s41927-021-00236-w.

## Background

Depression is a widely prevalent, chronic mood disorder that currently affects 322 million people worldwide [1]. The symptoms of depression can include depressed mood, lack of interest or pleasure in daily activities, weight loss, sleep disturbances, psychomotor issues, lack of energy, feelings of worthlessness or guilt, difficulty concentrating, and suicidal thoughts or actions [2, 3]. The severity of depression is determined by the number, intensity, and frequency of these symptoms, making it a heterogeneous disorder [3]. These symptoms significantly interfere with daily life and have made depressive disorders a leading cause of disability worldwide [4].

Depression is a common comorbidity of many chronic conditions, including rheumatoid arthritis (RA), and patients with depression and a chronic disease have worse health outcomes than those with either condition alone [5, 6]. A meta-analysis that included 72 studies of 13,189 patients with RA revealed that the prevalence of major depression in this population varies between 14.8% and 38.8%, depending on how symptoms are defined and measured [6]. This is much higher than the prevalence of depression in the general population, as just 7.1% of adults in the United States (US) reported that they experienced a major depressive episode in 2017 [7].

Social determinants of health (SDH) are conditions present in the environments where people are born, live, learn, work, play, worship, and age that influence people’s health, functioning, and quality of life outcomes [8]. Several studies have demonstrated that higher levels of food insecurity, defined as a household-level economic and social condition of limited or uncertain access to adequate food, are associated with worse mental health, especially in women [9–12]. Despite this link, little is known about the association between food insecurity and other SDH and depression among adults with RA. Given the elevated prevalence of depression in people with RA and the possible influence SDH have on depression in RA patients, investigating the association between depression and food insecurity in this population could help to identify new strategies for improving the mental health of these individuals. The objective of this study was to assess the prevalence of food insecurity and depression in adults with RA in the US noninstitutionalized population, and to assess the association between depression and SDH (e.g., food insecurity), demographics, and behavioral and lifestyle characteristics among adults with RA.

## Methods

### Data source

The National Health and Nutrition Examination Survey (NHANES) is a cross-sectional, nationally representative survey of the US Census civilian noninstitutionalized population [13]. The NHANES is a unique survey that uses interviews and physical examinations to assess the health and nutritional status of adults and children in the US. About 5000 people have been surveyed every year since 1999 and data are publicly released in 2-year cycles.

NHANES 2013–2014 and 2015–2016 were used in this study. Overall response rates among interviewed participants were 71% in NHANES 2013–2014 and 61.3% in NHANES 2015–2016; participants were asked about demographics, behavioral and lifestyle characteristics (e.g., smoking, alcohol, physical activities), SDH (e.g., food insecurity, household income), and disease history (e.g., whether they have been told by health professionals they have arthritis, which type of arthritis they have). The physical examination component takes place in a mobile examination center (MEC) and consists of medical, dental, and physiological measurements, as well as laboratory measurements.

### Study sample

To estimate the prevalence of depression and food insecurity among adults with RA, participants ≥ 18 years of age with self-reported RA (i.e., had been “told by doctor or other health professional” that they had “RA”) were selected from NHANES 2013–2014 and 2015–2016. To assess the association between depression and SDH as well as patients’ demographics, and behavioral and lifestyle characteristics, participants were required to have complete responses to the Patient Health Questionnaire-9 (PHQ-9) mental health-depression screener.

### Study measures

**Fig. 1:**
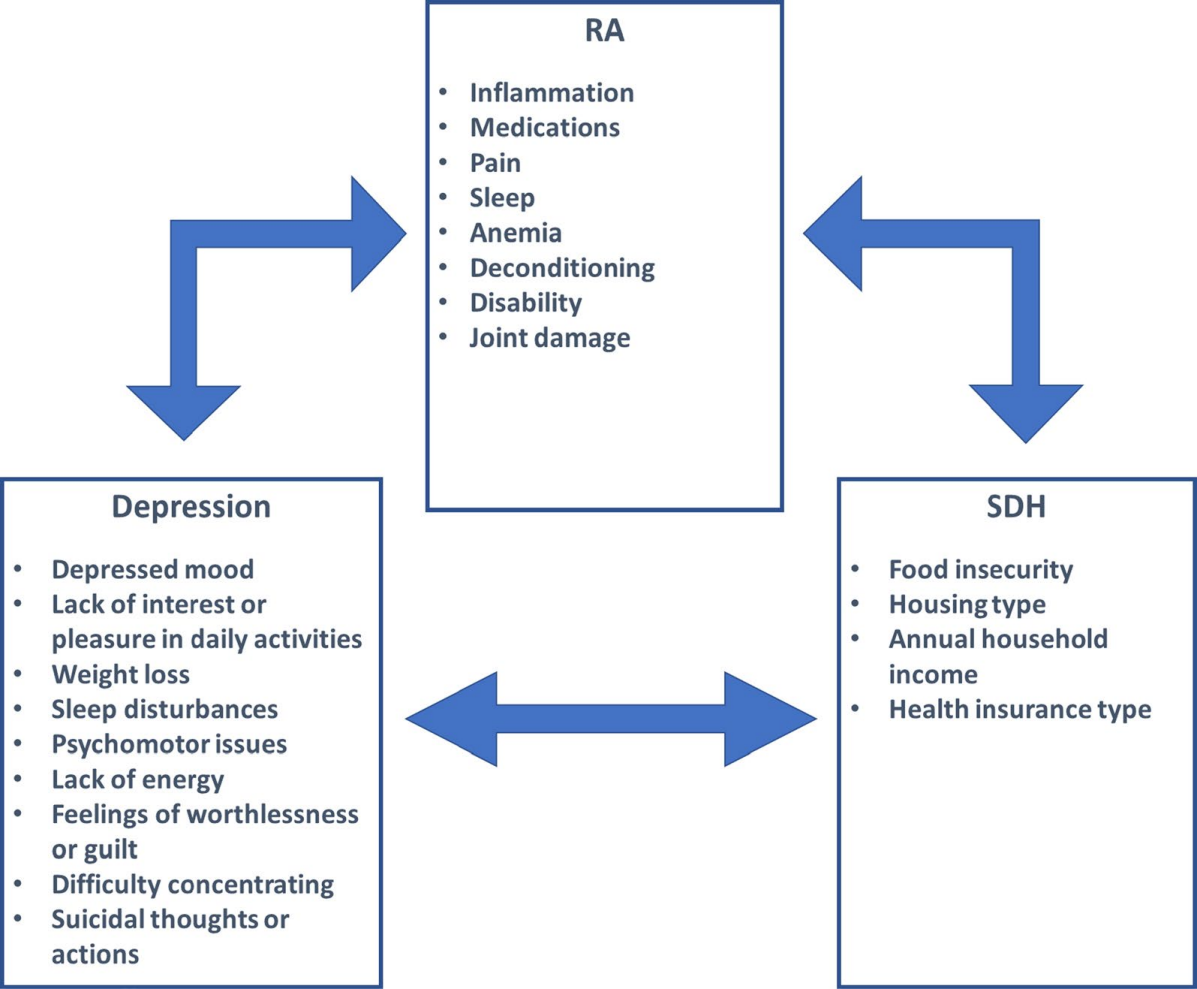
Proposed interactions between RA, depression, and SDH [2, 14, 15]. RA, rheumatoid arthritis; SDH, social determinants of health

A proposed model showing the interactions between RA, depression and SDH is shown in Fig. 1.

The PHQ-9, a 9-item depression screening instrument, was administered to measure the severity of depression symptoms patients experienced over the past 2 weeks [16, 17]. The psychometric properties of the PHQ-9, including internal consistency, and convergent and discriminant validity, have been examined in several studies of people with RA [18–20] and the PHQ-9 has been validated as a reliable diagnostic tool for depression in this population. The PHQ-9 incorporates the Diagnostic and Statistical Manual of Mental Disorders (DSM)-IV depression diagnostic criteria [21]. Response categories for the 9-item instrument are given a point value ranging from 0 to 3 based on the following responses: not at all, several days, more than half the days, and nearly every day. The sum of PHQ-9 scores, ranging from 0 to 27, was categorized as no/minimal depression (score of 0–4) or depression (score of ≥ 5). Depression was further stratified into mild depression (score of 5–9) and moderate-to-severe depression (score of 10–27), as used in previous studies, to describe the association between depression severity and other variables [22, 23].

Food insecurity was measured using the 18-item US Household Food Security Survey Module, in which the adult interviewee answered for the entire household [24]. To establish this survey, food security data taken annually from the Current Population Survey (CPS) was used to create a food security scale and a related categorical food-security-status measure to describe the food security of US households over the preceding 12 months. The stability and robustness of these measures were tested and validated in the overall population across years and major population groups. A raw score was created by summing the affirmative responses to the 18 questions, and a categorical variable was created to characterize the overall food security status for the entire household as (1) full food security (no affirmative response to any item); (2) marginal food security (1–2 affirmative responses); (3) low food security (3–7 affirmative responses); and (4) very low food security (8–18 affirmative responses) [12, 25]. As an objective of this study was to examine the prevalence of different levels of food insecurity among adults with RA, food insecurity was defined as any category other than full food security (i.e., marginal, low, or very low food security).

Other SDH assessed in this study included health insurance type, education, marital status, annual household income, number of members in household, and housing characteristics. Key behavioral and lifestyle characteristics were determined using information provided by participants on their general health condition, alcohol use, smoking status, physical disability status (i.e., whether or not to have serious difficulty hearing, seeing, concentrating, walking, dressing, or running errands), body mass index, history of overnight hospitalization, work/recreational activity level, number of physical limitations, sleep disorder status, and attempts to lose weight.

### Statistical analyses

To make inference on population parameters and obtain representative estimates of the US noninstitutionalized population, all sample data were weighted to account for different sampling probabilities, non-response rates, stratification, and clustering of observations introduced by the NHANES’ complex survey design [26]. As depression was assessed during the interview at the MEC from 2013 to 2016, the 4-year MEC exam weight was used in this study.

The weighted prevalence of depression and food insecurity in US adults with RA were estimated using SAS survey procedures. Unadjusted prevalence was reported for the 2013–2014 and 2015–2016 waves and odds ratios (ORs) and 95% confidence intervals (CIs) were calculated to assess temporal changes in the prevalence of depression and food insecurity over 4 years (i.e., 2 NHANES waves).

SDH, demographics, and behavioral and lifestyle characteristics were described and compared between RA patients with depression and those with no/minimal depression. Univariable logistic regression models were used to evaluate the association between depression and individual factors. Unadjusted ORs and 95% CIs were calculated. Penalized regression with least absolute shrinkage and selection operator (LASSO) method was performed to select the variables for the final multivariable model [27]. Penalized regression using the LASSO method has become a preferred method for variable selection because it can reduce data dimensionality by shrinking a subset of the coefficients to 0 and reduce the complexity of the model. The association between the selected factors and depression was finally evaluated by fitting a weighted multivariable logistic regression model, and results were presented as adjusted ORs and 95% CIs.

All analyses were performed with SAS (Version 7.1, SAS Institute Inc., Cary, North Carolina) and R (Version 3.6.3, R Foundation for Statistical Computing, Vienna, Austria) software.

## Results

**Fig. 2:**
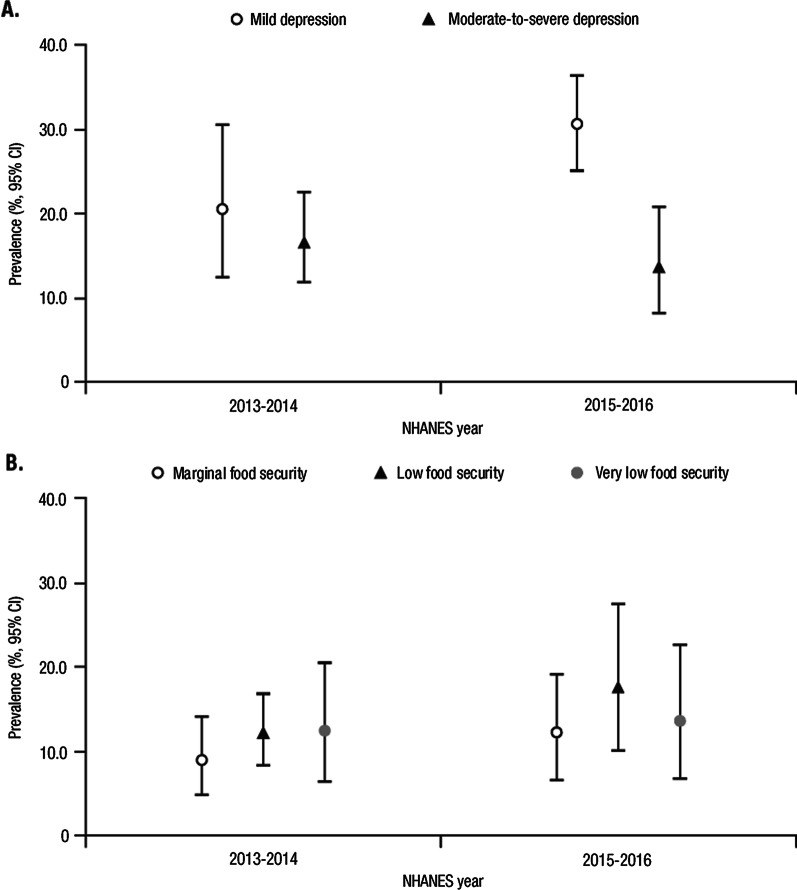
Weighted prevalence of **A** depression and **B** food insecurity among adults with RA. RA, rheumatoid arthritis; CI, confidence interval; NHANES, National Health and Nutrition Examination Survey

NHANES 2013–2014 included 10,175 participants and NHANES 2015–2016 included 9,971 participants. Of these, a total of 251 adult respondents from 2013 to 2014 and 276 from 2015 to 2016 had self-reported RA. Among the 527 respondents with RA, 90.1% (*n* = 475) completed the PHQ-9. Overall, the prevalence of depression was 37.1% in 2013–2014 and 44.1% in 2015–2016 among adults with RA; there was an apparent shift in the prevalence of mild depression from 20.4% in 2013–2014 to 30.5% in 2015–2016, although we cannot rule out the possibility of between-cycle sampling variation influencing these estimates (Fig. 2A). Similarly, the prevalence of food insecurity was 33.1% in 2013–2014 and 43.0% in 2015–2016 among adults with RA (Fig. 2B). Although no significant difference was detected in the prevalence of marginal, low, and very low food security, there appeared to be an increase in all categories of food insecurity from 2013–2014 to 2015–2016.

Demographics, and behavioral and lifestyle characteristics of study participants with depression compared with those with no/minimal depression are presented in Table 1. Compared to adults with RA and no/minimal depression, those with depression were generally more likely to be aged 45–64 years, be female, be a current smoker, report fair/poor health condition, report having a physical disability, or have ≥ 4 physical limitations.

**Table 1:** Demographics, and Behavioral and Lifestyle Characteristics of Participants with RA*

	No/minimal depression (*n* = 268)	Depression (*n* = 207)	OR (95% CI)
Age, years			
18–44	43 (20.6)	25 (15.2)	1.09 (0.52, 2.25)
45–64	104 (38.1)	106 (56.9)	2.20 (1.23, 3.96)
65+	121 (41.3)	76 (28.0)	Reference
Gender			
Male	126 (43.0)	64 (31.7)	Reference
Female	142 (57.0)	143 (68.3)	1.62 (1.02, 2.58)
Race/ethnicity			
Non-Hispanic White	108 (65.6)	76 (65.3)	Reference
Non-Hispanic Black	76 (16.5)	46 (13.1)	0.80 (0.46, 1.38)
Hispanic	65 (12.3)	74 (17.6)	1.44 (0.84, 2.49)
Other	19 (5.6)	11 (4.1)	0.72 (0.27, 1.97)
Self-reported general health condition			
Excellent/very good/good	183 (74.5)	68 (39.6)	Reference
Fair/poor	85 (25.5)	139 (60.4)	4.47 (2.56, 7.80)
Alcohol use			
None/moderate drinker^†^	211 (75.3)	149 (64.9)	Reference
Heavy/binge drinker	57 (24.7)	58 (35.1)	1.66 (0.72, 3.81)
Smoking status			
Never	130 (50.0)	87 (35.3)	Reference
Former	87 (32.3)	53 (32.7)	1.43 (0.72, 2.86)
Current	49 (17.2)	67 (32.0)	2.64 (1.42, 4.89)
Not reported	2 (0.4)	0 (0.0)	–
Any physical disability	119 (42.1)	160 (79.3)	5.27 (2.83, 9.80)
BMI			
Normal (< 25 kg/m^2^)	54 (22.3)	19 (12.7)	Reference
Overweight (25– < 30 kg/m^2^)	80 (29.3)	63 (28.5)	1.71 (0.53, 5.46)
Obese (≥ 30 kg/m^2^)	130 (47.5)	118 (55.6)	2.05 (0.67, 6.33)
Not reported	4 (0.9)	7 (3.2)	–
Overnight hospitalization in last year	44 (15.9)	58 (22.6)	1.54 (0.73, 3.24)
Moderate or vigorous work activity	118 (48.3)	78 (43.8)	0.83 (0.55, 1.28)
Moderate or vigorous recreational activity	108 (43.3)	56 (29.3)	0.54 (0.32, 0.94)
Number of physical limitations^‡^			
0–3	143 (55.4)	41 (18.4)	Reference
≥ 4	125 (44.6)	166 (81.6)	5.50 (3.06, 9.89)
Not reported			
Sleep disorder^§^	22 (22.9)	25 (27.8)	1.30 (0.50, 3.39)
Attempt to lose weight in last year	81 (32.4)	80 (39.1)	1.33 (0.75, 2.38)
Not reported	53 (20.6)	35 (18.4)	–

RA, rheumatoid arthritis; OR, odds ratio; CI, confidence interval; BMI, body mass index; NHANES, National Health and Nutrition Examination Survey

^*^Values are n (weighted %)

^†^ ≤ 1 drink/day for females, ≤ 2 drinks/day for males

^‡^A physical functioning index will be created by summing scores for all 20 questions designed to assess difficulty in performing a variety of functional activities without the aid of special equipment and categorized into 3 groups: No physical limitations, 1–3 physical limitations, 4+ physical limitations

^§^Responses to questions about sleep disorder were only collected in the 2013–2014 NHANES and the association between sleep disorder and depression was assessed among 224 adults with available data

Participants with RA who reported having food insecurity had about twofold higher odds of experiencing depression than participants with full food security (OR 2.17, 95% CI 1.27, 3.72; Table 2); though the directionality of associations could not be determined. This association was particularly pronounced for those with very low food security (OR 2.96, 95% CI 1.48, 5.90). An increased odds of food insecurity was noted with more severe depression (Additional file 1: Table 1). The odds of having mild depression and moderate-to-severe depression were 2.03 times (95% CI 1.07, 3.86) and 2.44 times (95% CI 1.10, 5.43) higher, respectively, for participants who reported food insecurity than those who reported full food security.

**Table 2:** SDH of adults with RA*

	No depression (*n* = 268)	Depression (*n* = 207)	OR (95% CI)
Health insurance type			
Private	129 (55.3)	70 (40.8)	Reference
Non-private (Medicare/medicaid/other)	117 (37.0)	105 (46.6)	1.71 (0.96, 3.04)
Not reported	22 (7.8)	32 (12.5)	
Education level			
Less than college education	137 (43.4)	127 (48.7)	Reference
Any college or higher education	131 (56.6)	80 (51.3)	0.81 (0.56, 1.16)
Marriage status			
In a marriage/partnership	154 (61.9)	98 (50.9)	Reference
Not in a marriage/partnership	114 (38.1)	109 (49.1)	1.56 (0.94, 2.60)
Annual household income			
< $20,000	77 (19.9)	75 (26.9)	1.46 (0.88, 2.40)
≥ $20,000	181 (77.5)	129 (72.1)	Reference
Not reported	10 (2.6)	3 (1.0)	–
Number of members in household (median, IQR)	1.74 (1.17, 3.10)	1.85 (1.16, 3.13)	1.02 (0.90, 1.15)
Housing type			
Owned/bought	188 (76.5)	115 (62.9)	Reference
Rented/other management	79 (23.4)	91 (36.8)	1.91 (1.06, 3.47)
Not reported	1 (0.1)	1 (0.3)	
Food insecurity			
No	168 (69.0)	94 (50.5)	Reference
Yes	100 (31.0)	113 (49.5)	2.17 (1.27, 3.72)
Marginal food security	34 (9.5)	28 (11.7)	1.69 (0.73, 3.93)
Low food security	41 (12.5)	44 (18.1)	1.98 (0.88, 4.45)
Very low food security	25 (9.1)	41 (19.6)	2.96 (1.48, 5.90)

SDH, social determinants of health; RA, rheumatoid arthritis; OR, odds ratio; CI, confidence interval; IQR, interquartile range

^*^Values are n (%) unless noted otherwise

In addition to food insecurity, other SDH such as housing type were associated with depression in participants with RA. Adults who rented their home had a higher likelihood of experiencing depression compared with those who owned their home (OR 1.91, 95% CI 1.06, 3.47). Furthermore, stratified analyses showed that participants with an annual household income < $20,000 were more likely to have moderate-to-severe depression than those with an annual household income ≥ $20,000 (OR 2.22, 95% CI 1.26, 3.90). Participants with insurance through non-private plans were more likely to have moderate-to-severe depression than those with private insurance (OR 2.17, 95% CI 1.05, 4.52).

**Fig. 3:**
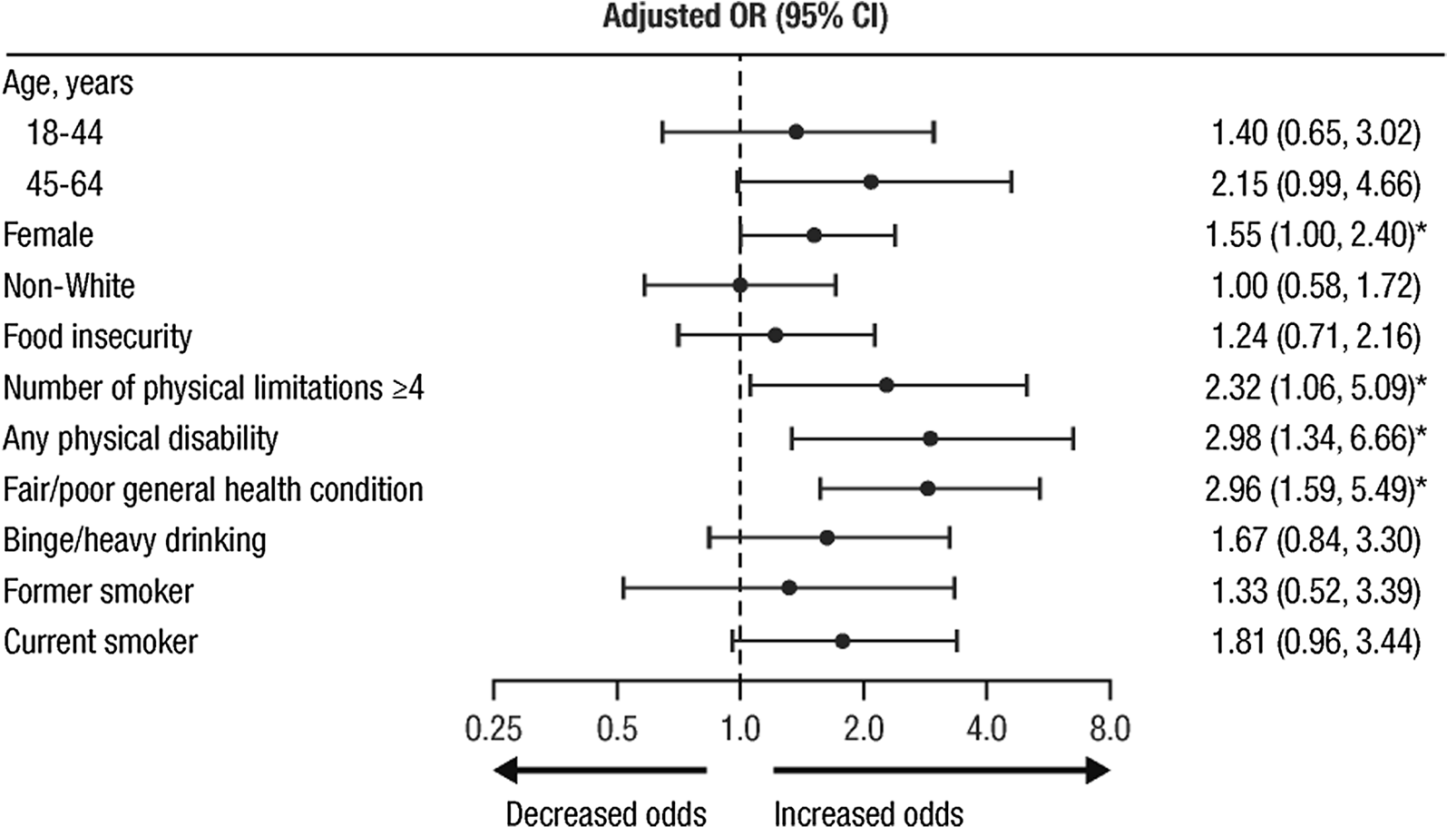
Association between depression and selected variables among participants with RA. RA, rheumatoid arthritis; OR, odds ratio; CI, confidence interval. *OR (95% CI) indicates statistically significant difference

Multivariable logistic regression showed that being female, being in fair/poor general health, having a physical disability (in terms of hearing, seeing, concentrating, walking, dressing, or running errands), and having ≥ 4 physical limitations in performing a variety of functional activities were significantly associated with depression in participants with RA (Fig. 3).

## Discussion

To our knowledge, this is the first study to provide prevalence estimates of depression and food insecurity for US adults with RA. This study found that depression was associated with food insecurity among adults with RA and this association varied by depression severity, with larger effects observed among adults with moderate-to-severe depression than those with mild depression, though the directionality of these associations could not be determined.

A previous study that used 2005–2008 NHANES data estimated that the prevalence of depression was 21.6% in the general US adult population [28]. Our study found that the prevalence of depression among adults with RA was 37.1% and 44.1% in 2013–2014 and 2015–2016, respectively, which is nearly double the prevalence observed in the general population. While depression is more common in people with chronic conditions compared with the general population, the prevalence of depression is relatively higher in adults with RA than those with other chronic conditions such as diabetes and lung disease [29]. Specifically, data from this study revealed that 13.7%-16.6% of RA participants reported having moderate-to-severe depression, which is a greater proportion than in the general US population (6.6–6.8%) [28, 30].

Food insecurity may increase an individual’s dependence on inexpensive energy-dense foods that lead to the development of chronic diseases that may affect mental health, and the risk of developing chronic conditions increases as food security diminishes [31, 32]. Previous studies have shown that food insecurity was associated with high risk of chronic diseases, such as hypertension, asthma, and chronic obstructive pulmonary disease [31–33]. In this study, unadjusted analyses showed that adults with RA and food insecurity were more likely to report depression, with the greatest effects seen in those with very low food security, who were approximately 3 times more likely to experience depression than participants without food insecurity. In unadjusted analyses, a stronger relationship between food insecurity and depression was observed in those with more severe depression. However, after adjustment for other covariates, food insecurity was not shown to have a significant association with depression.

Previous studies have shown that food insecurity and other SDH are highly correlated with behavioral and lifestyle characteristics [34, 35]. Thus, the effect of food insecurity on depression in the multivariate analysis may have been diluted by other strong prognostic covariates. In addition to food insecurity, this study showed that other SDH such as living in a rented home were associated with depression in participants with RA. Other studies on the impact of SDH on depression in patients with RA have found correlations between a variety of socioeconomic factors, such as low socioeconomic status, urban residence, public clinic use, and low levels of social support, and depression in this population [36–39].

Several demographic and behavioral characteristics like being female, having fair/poor health condition, current smoking, having a physical disability, and having serious functional limitations were also significantly associated with depression in participants with RA. Previous reports have shown that RA progression may be associated with an increased risk of depression [40]. A systematic review reports smoking is associated with depression although the direction of the association is not clear [41]. Findings from this study add to the body of literature showing a positive association between current smoking and depression among adults with RA.

There are several limitations that should be considered when interpreting these findings. As with any database study, analyses were limited to the variables that were collected. NHANES data are self-reported and do not contain comprehensive details such as physician diagnosis of conditions (e.g., depression, RA), measures of disease severity of activity, or prescription use (e.g., antidepressants, biologics). Although some medication use has been captured in NHANES as an exploratory measure, antidepressant use was only collected in NHANES 2015–2016, and only 9 out of 475 respondents reported using biologics at any time during the study period, suggesting a large amount of missing data. As in all health surveys, the accuracy of the data collected depends on respondents’ truthfulness and memory, so it is subject to misclassification, non-response, and recall bias. The NHANES is designed as a cross-sectional study; therefore, findings are limited to assess the directionality and causality of the relationship between depression and SDH including food insecurity. Previous results suggest that there is a bidirectional relationship between food insecurity and poor emotional health [15]; however, more comprehensive studies are needed to understand responsible factors. Although the NHANES questionnaire is considered a highly reliable measure of health status in the US population, it excludes institutionalized individuals and those in the military, which limits the generalizability of these results. Additionally, the number of participants with RA was relatively limited and studies with larger numbers of participants could be used to further assess associations between SDH and depression severity.

Despite these limitations, NHANES is a nationally representative database that contains participants selected to represent the entire US population regardless of insurance status. This feature is unique compared to several other patient databases and studies thereof. Additionally, the NHANES is conducted using standardized procedures, limiting variability due to inconsistencies in survey execution.

## Conclusions

Findings from this study showed that the prevalence of depression and food insecurity were high in adults with RA. Unadjusted analyses showed that participants with self-reported RA were more likely to suffer from depression if they were experiencing food insecurity, though this association was not significant when adjusted for behavioral/lifestyle characteristics. Providing supportive resources to address those SDH amenable to change may help manage the burden of depression among adults with RA. These results warrant further investigation into the relationship between depression and SDH in people with RA.

## Supplementary Information

**Additional file 1:** SDH of Adults with RA by Depression Severity.

## Data Availability

The datasets supporting the conclusions of this article are available in the NHANES repository, https://www.cdc.gov/nchs/nhanes/about_nhanes.htm.

## Abbreviations

BMI: Body mass index
CI: Confidence interval
CPS: Current Population Survey (CPS)
DSM: Diagnostic and Statistical Manual of Mental Disorders
IQR: Interquartile range
LASSO: Least absolute shrinkage and selection operator
MEC: Mobile examination center
NHANES: National Health and Nutrition Examination Survey
OR: Odds ratio
PHQ-9: Patient Health Questionnaire-9
RA: Rheumatoid arthritis
SDH: Social determinants of health
US: United States
