# Supplementary material for: Depression and food insecurity among patients with rheumatoid arthritis in NHANES
Source: BMC Rheumatol. 2022 Feb 2;6:6. doi: 10.1186/s41927-021-00236-w (PMC8808967; doi:10.1186/s41927-021-00236-w)
Supplement: Supplementary file 1 — Additional file 1: SDH of Adults with RA by Depression Severity. [file 41927_2021_236_MOESM1_ESM.docx]

**Depression and Social Determinants of Health Among Patients with Rheumatoid Arthritis in NHANES**

Qian Cai^1^, Jacqueline Pesa^1^, Ruibin Wang^1,2^, Alex Z. Fu^1,3^

^1^Janssen Scientific Affairs, LLC, Titusville, NJ, USA.

^2^Harvard TH Chan School of Public Health, Cambridge, MA, USA.

^3^Georgetown University Medical Center, Washington, DC, USA.

**Supplemental Table 1. SDH of Adults with RA by Depression Severity**

| Characteristic* | Mild depression (PHQ-9: 5-9) (*n* = 123) | OR (95% CI) | Moderate-to-severe depression (PHQ-9: 10+) (*n* = 84) | OR (95% CI) |
| --- | --- | --- | --- | --- |
| Health insurance type |  |  |  |  |
| Private | 47 (45.1) | reference | 23 (33.7) | reference |
| Medicare/Medicaid/Other | 57 (45.2) | 1.50 (0.73, 3.09) | 48 (49.0) | 2.17 (1.05, 4.52) |
| Not reported | 19 (9.7) |  | 13 (17.3) |  |
| Education level |  |  |  |  |
| Less than college education | 73 (42.4) | reference | 54 (59.3) | reference |
| Any college or higher education | 50 (57.6) | 1.04 (0.66, 1.65) | 30 (40.7) | 0.53 (0.27, 1.01) |
| Marriage status |  |  |  |  |
| In a marriage/partnership | 63 (49.8) | reference | 35 (52.8) | reference |
| Not in a marriage/partnership | 60 (50.2) | 1.64 (0.90, 2.99) | 49 (47.2) | 1.45 (0.78, 2.68) |
| Not reported |  |  |  |  |
| Household income bracket |  |  |  |  |
| <$20,000 | 40 (21.7) | 1.09 (0.59, 2.01) | 35 (35.6) | 2.22 (1.26, 3.90) |
| ≥$20,000 | 82 (77.7) | reference | 47 (62.7) | reference |
| Not reported | 1 (0.6) |  | 2 (1.6) |  |
| Number of members in household (median, IQR) | 1.90 (1.23, 3.25) |  | 1.75 (1.05, 2.90) |  |
| Housing type |  |  |  |  |
| Owned/bought | 68 (61.2) | reference | 47 (65.6) | reference |
| Rented/other management | 54 (38.3) | 2.05 (1.05, 3.97) | 37 (34.4) | 1.71 (0.78, 3.76) |
| Not reported | 1 (0.5) |  | 0 |  |
| Food insecurity |  |  |  |  |
| No | 57 (52.3) | reference | 37 (47.6) | reference |
| Yes | 66 (47.7) | 2.03 (1.07, 3.86) | 47 (52.4) | 2.44 (1.10, 5.43) |

SDH, social determinants of health; RA, rheumatoid arthritis; PHQ-9, Patient Health Questionnaire-9; OR, odds ratio; CI, confidence interval; IQR, interquartile range.

*Values are n (%) unless noted otherwise.
